# Sleep Health and Quality of Life in Children and Adolescents with NF1: A Biopsychosocial Perspective

**DOI:** 10.3390/cancers18142366

**Published:** 2026-07-22

**Authors:** Natalie A. Pride, Siobhan Banks, Dinberu Shebeshi, Shelley S. Arnold, Kristina Haebich, Jessica Habib, Crystal Yates, Hayley Darke, Kathryn N. North, Jack Nguyen, Jonathan M. Payne

**Affiliations:** 1 Kids Neuroscience Centre, Sydney Children’s Hospital Network, Westmead, NSW 2145, Australia; 2 Sydney Medical School, The University of Sydney, Camperdown, NSW 2050, Australia; 3 Behaviour Brain Body Research Centre, Adelaide University, Adelaide, SA 5001, Australia; 4 Kids Research, Sydney Children’s Hospital Network, Westmead, NSW 2145, Australia; 5 Murdoch Children’s Research Institute, Parkville, VIC 3052, Australia; 6 Department of Paediatrics, Faculty of Medicine, Dentistry and Health Sciences, University of Melbourne, Parkville, VIC 3052, Australia

**Keywords:** neurofibromatosis 1, sleep, biopsychosocial, neurodevelopmental, sex, quality of life

## Abstract

**Simple Summary:**

Preclinical evidence suggests that the *NF1* gene is important for sleep regulation and circadian health. This study applies Buysse’s sleep health framework to characterize sleep in children and adolescents with neurofibromatosis type 1 (NF1) and to examine its relationship with biopsychosocial factors and quality of life (QoL). We found that poorer sleep health in NF1 was associated with male sex, elevated pain, and neurodevelopmental traits and was characterized by impaired sleep efficiency, daytime sleepiness, insufficient sleep duration, poor subjective sleep quality, and pre-bedtime hyperarousal. Sleep duration independently predicted QoL in NF1. These findings inform the development of targeted multimodal interventions to improve sleep health in children and adolescents with NF1.

**Abstract:**

Background: This study applies Buysse’s sleep health framework to examine sleep in children and adolescents with neurofibromatosis type 1 (NF1). By examining sleep timing, daytime sleepiness, sleep quality, sleep behavior, sleep duration, and sleep efficiency together, this framework captures the multidimensional nature of sleep and its relationship with biopsychosocial factors and health-related quality of life (HR-QoL) in NF1. Methods: This multi-site, prospective, cross-sectional study included 131 children and adolescents with NF1 and 71 typically developing (TD) controls aged 6 to 16 years. A sleep health composite was derived from carer rating scales and 7 days of actigraphy. A biopsychosocial framework was used to examine factors associated with sleep health in NF1, including sociodemographic, cognitive, psychopathology, and biological variables. Independent predictors of QoL were examined to assess the unique contributions of sleep quality, sleep duration, and previously established predictors of HR-QoL in NF1. Results: Poorer sleep health was evident in children with NF1. Compared with TD controls, children with NF1 were five times as likely to have poor sleep quality, with almost 78% demonstrating impaired sleep efficiency and nearly half not obtaining sufficient sleep at night. The strongest risk factors were being male, elevated pain, and having greater levels of ADHD and autism spectrum disorder traits. Conclusions: Findings suggest sleep health in NF1 is interconnected with multiple biopsychosocial factors. A better understanding of these relationships will help identify early risk markers, improve prediction of clinical trajectories, and guide the development of targeted multimodal interventions for sleep disruption in NF1.

## 1. Introduction

Neurofibromatosis type 1 (NF1) is a genetic condition that results from a pathogenic variant in the *NF1* gene encoding neurofibromin. This protein regulates cell growth, division, and differentiation, primarily by acting as a GTPase-activating protein (GAP) that negatively regulates RAS activity [1]. NF1 is associated with a broad range of medical complications, including tumors of the peripheral and central nervous systems (CNS), musculoskeletal changes, and neurological involvement [2]. Cognitive deficits and an increased risk of neurodevelopmental disorders, including attention deficit hyperactivity disorder (ADHD), autism spectrum disorder (hereafter referred to as autism), and specific learning disabilities, are all common and collectively may contribute to functional impairment and reduced quality of life (QoL) [3,4,5].

Studies across a range of *Nf1* animal models, including heterozygous and conditional knockouts, demonstrate that the *NF1* gene is required for normal regulation of sleep–wake behavior. In the *Drosophila* knockout model, a short sleep phenotype [6], altered circadian rhythm [7,8], and sleep fragmentation [6] have been observed. Lower arousal threshold and/or increased sleep fragmentation have also been demonstrated in other heterozygous models of NF1, including zebrafish [9], mice [10], and minipig [11]. Overall, this evidence suggests that the *NF1* gene is important for sleep–wake regulation. Characterizing sleep problems in humans with NF1, however, is challenging. This is not only because sleep is multidimensional but also because NF1 is a heterogeneous multisystem condition in which sleep disturbance likely reflects a complex interplay of biological, psychological, and sociodemographic factors.

The few studies that have examined sleep in children and adolescents (hereafter referred to as children) with NF1 have relied on subjective measures (self-report or caregiver questionnaires), often only capturing a single sleep construct. Findings regarding the type of sleep disturbance in NF1 are inconsistent. Johnson et al. [12], using the Simonds and Parraga questionnaire, reported elevated parasomnia in NF1 relative to normative data, whereas Licis et al. [13], using the Sleep Disturbance Scale for Children, reported greater problems initiating and maintaining sleep, arousal difficulties, sleep–wake transition disorders, and hyperhidrosis in NF1 compared with unaffected siblings. More recently, objective assessment using actigraphy has demonstrated longer sleep onset latency, poorer sleep efficiency and regularity, shorter sleep duration, and greater night-to-night variability in sleep timing in children with NF1 compared with typically developing (TD) controls [14]. These inconsistencies likely reflect the multidimensional nature of sleep health. Studies capturing different constructs in isolation will naturally yield different findings.

Buysse (2014) posits sleep health as a multidimensional construct rather than a unidimensional measure or the absence of a sleep disorder [15]. It is defined as “a multidimensional pattern of sleep-wakefulness, adapted to individual, social, and environmental demands, that promotes physical and mental wellbeing.” Buysse conceptualized sleep as a positive attribute that contributes to the overall health and functioning of an individual or population [15]. This framework, which has been recently adapted to paediatrics [16], identifies six dimensions of sleep critical to psychological and physical wellbeing: sleep duration, sleep efficiency, sleep timing, daytime sleepiness, sleep quality, and sleep-related behaviors (pre-sleep activities compatible with sleep). Meaningful impairment can occur across these domains without meeting formal diagnostic criteria, underscoring the need for multidimensional assessment of sleep health in both clinical and research contexts.

Taken together, existing findings indicate that sleep difficulties are common in NF1, yet their nature and determinants remain poorly understood. A biopsychosocial framework offers a more integrative approach. Within this framework, poor sleep may arise from (1) intrinsic biological differences related to *NF1*-associated alterations in neurobiological networks; (2) psychosocial factors such as depression, anxiety, pain, cognitive processing, and behavioral and emotional dysregulation; and (3) contextual factors such as socioeconomic status (SES), parenting practices, and technology use [17]. For example, emerging evidence indicates that biological alterations are associated with disrupted sleep in children with NF1, including changes in endogenous melatonin secretion [14] and sleep architecture [18]. Research examining links between sleep and the physical manifestations of NF1, however, remains limited. Chronic pain is common in NF1—often related to plexiform neurofibromas, skeletal complications, and headaches [3]—and is a plausible contributor to sleep disruption. Clarifying the sleep–pain relationship in children with NF1 is clinically important, given evidence of bidirectional effects [19]. Recent studies also suggest associations between sleep disturbance and ADHD symptoms in NF1 [14,20]. To date, no study has integrated neurodevelopmental traits, cognition, social–emotional symptoms, and pain within a single biopsychosocial study to examine their unique associations with multidimensional sleep health in NF1.

Because the same biopsychosocial factors that we hypothesize are associated with sleep in NF1 (e.g., pain, neurodevelopmental traits, mental health symptoms) also have well-established links to functioning and health-related quality of life (HR-QoL) [4,21,22], it is important to determine whether sleep health explains unique variance in HR-QoL beyond these established predictors. In the general population, sleep duration and sleep quality are consistently linked to QoL [23,24,25], such that shorter sleep and poorer sleep quality are linked to reduced physical, emotional, and social functioning. Given that sleep difficulties are amenable to intervention, understanding their contribution to functional outcomes and QoL in NF1 has direct clinical relevance. To date, however, no study has examined this relationship.

This study had three aims. First, we aimed to describe multidimensional sleep health characteristics in children with NF1 and compare these domains to a typically developing (TD) control group. Second, we examined the associations between sleep health and biopsychosocial factors in NF1. Third, we examined whether sleep quality and duration predicted HR-QoL alongside established predictors of HR-QoL in children with NF1.

## 2. Materials and Methods

### 2.1. Participants and Procedures

**Figure 1:**
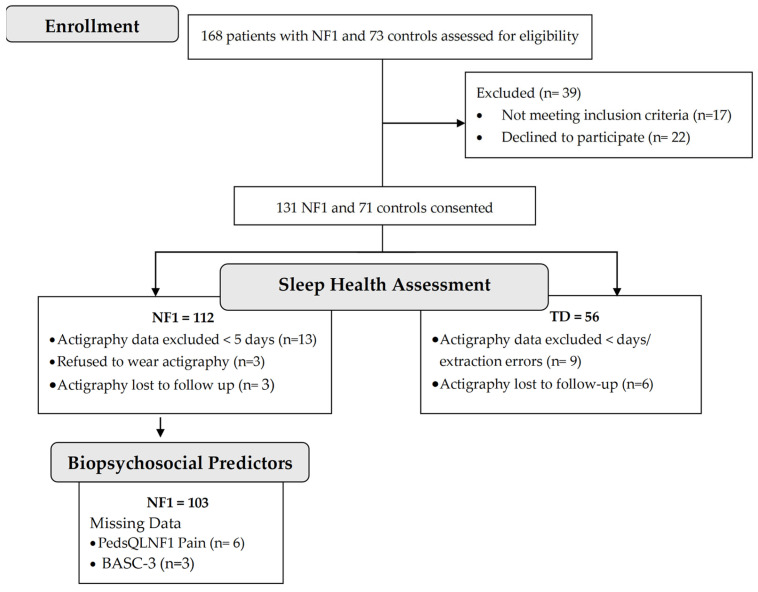
CONSORT flow diagram of participant recruitment and inclusion.

Participants were 131 children and adolescents with NF1 recruited through outpatient clinics at The Children’s Hospital at Westmead in Sydney and the Royal Children’s Hospital in Melbourne. Children aged 6–16 years who met diagnostic criteria for NF1 were included. Those who had received chemotherapy or radiation therapy in the prior 12 months were excluded. A TD control group aged 6–16 years (*n* = 71) was recruited through research and community networks. Controls with a history of developmental delay, neurodevelopmental disorder, or neurological or psychological conditions were excluded. Eligible participants completed a neuropsychological assessment, and caregivers completed surveys and questionnaires. Participants were also asked to wear a wrist-worn accelerometer that measures rest/activity cycles for 1 week and complete a sleep diary during the same week. Either the Actiwatch 2 (Philips Respironics, Bend, OR, USA) or the Geneactiv accelerometer (Activinsights Ltd, Kimbolton, UK) was provided, and data were scored using a standard algorithm that had been validated in paediatric populations and across the two devices [26,27]. Participants with fewer than 5 days of data were excluded. Group differences on individual actigraphy-derived outcomes (sleep efficiency, total sleep time, sleep timing) from this dataset have been reported on previously [14]. Here, we adopted a conceptually distinct approach by dichotomizing key sleep health measures, consisting of objective actigraphy data and novel subjective questionnaire data, aggregating them into a composite sleep health score in accordance with the paediatric sleep health framework [16]. The study was approved by the Sydney Children’s Hospital Network Ethics Committee. Written informed consent was obtained from all participants. Figure 1 shows the flow of participants into the study and participant exclusions. OpenAI (GPT-5.5) was used to assist in the creation of the graphical abstract following author developed prompts. The authors reviewed, edited, and verified the final figure. 

### 2.2. Measures

#### 2.2.1. Sleep Health Composite

We created a multidimensional sleep health composite score capturing six dimensions: sleep duration, sleep timing, sleep quality, daytime sleepiness, sleep efficiency, and sleep behaviors, as defined by the sleep health paediatric framework [16]. Each dimension was converted into a binary variable, where 1 represents an unfavorable sleep characteristic, and 0 indicates a favorable characteristic (see Table 1). Cutoffs were based on previous literature and informed by clinical and empirical cut points [28,29,30]. The sleep health composite required at least 5 of 6 components to be present. Prorated sums were then calculated, with higher scores indicating poorer sleep health. Further details regarding variable coding are provided in the Supplementary Materials (Table S1).

#### 2.2.2. Biopsychosocial Factors

Mental health was assessed using Depression and Anxiety Scale T scores from the Behavior Assessment System for Children Version 3 (BASC-3) Parent Rating Scale (PRS) [34], using either the child (6–11 years) or adolescent (12–21 years) form. Given Depression and Anxiety T scores were highly correlated in this NF1 cohort (*r* = 0.69), and to avoid multicollinearity, a composite measure of mental health was created by summing and averaging the T scores. Higher scores indicated poorer mental health.

Cognition was assessed with the Full-Scale Intelligence Quotient (FSIQ) from the Wechsler Intelligence Scale for Children 5th Version Australian and New Zealand Edition (WISC-V A&NZ) [35].

Neurodevelopmental traits were assessed using the ADHD Inattentive and ADHD Hyperactive/Impulsive subscale T scores from the Conners 3 Parent Questionnaire [36] and the Restricted and Repetitive Behaviors T Score and Social Interactions and Social Communication T Score from the Social Responsiveness Scale—Version 2 (SRS-2) [37]. Given the substantial overlap among these traits in NF1 [38], and to reduce multicollinearity (*r* = 0.65–0.88), these symptoms were combined into a single neurodevelopmental trait composite by summing and averaging the four T scores. Higher T scores indicated more elevated neurodevelopmental traits.

Biological variables included pain, assessed using the Parent Proxy measure of the Paediatric Quality of Life Neurofibromatosis Type 1 Module (PedsQL-NF1) [39]. Young child (5–7 years), child (8–12 years), and adolescent (13–18 years) versions were used. We selected the pain subscale, which has been well-validated in paediatric NF1 cohorts [40]. Scores range from 0 to 100, with lower scores indicating increased subjective pain. Physical complications of NF1 were reported by parents and classified as a skeletal abnormality (osseous lesion, scoliosis, or sphenoid wing dysplasia), one or more plexiform neurofibroma, an optic pathway glioma (OPG), or CNS abnormality (other than OPG, e.g., epilepsy, cerebral tumor, hydrocephalus). Medication use was also recorded and classified as ADHD medication or other CNS medications (e.g., anticonvulsants, antidepressants, analgesics).

Sociodemographic variables included age, sex, and socioeconomic status (SES), which were controlled for in all analyses. The Index of Relative Socio-Economic Advantage and Disadvantage from Socio-Economic Indexes for Areas, a standard measure that ranks postal codes based on average income, education, and occupational level, was used to assess SES [41]. Decile scores range from 1 to 100, with higher scores indicating higher SES.

#### 2.2.3. Health-Related Quality of Life

The Paediatric Quality of Life Inventory (PedsQL) Version 4.0 Generic Core Scales [42] was used to assess HR-QoL. The Total Scale Score, calculated as the mean of all 23 items, was used as overall generic HR-QoL. Scores range from 0 to 100, with higher scores indicating better HR-QoL. Parent proxy reports for children and adolescents were completed.

### 2.3. Data Analysis

All analyses were conducted using IBM SPSS Statistics software (Version 25) [43] or JASP (Version 0.96) [44]. Group differences in demographic and clinical characteristics between children with NF1 and TD controls were examined using independent *t*-tests for continuous variables or chi-square tests for categorical variables. Statistical analyses for all aims controlled for age, sex, and SES. For aim 1, an analysis of covariance (ANCOVA) was conducted to examine group differences in sleep health. Assumptions of ANCOVA were assessed prior to analysis, including normality, homogeneity of variance, and homogeneity of regression slopes. To account for minor violations of heteroscedasticity, bootstrapping with 5000 resamples was used to generate 95% confidence intervals and *p*-values. Interaction terms between groups and each covariate were included to test the assumption of homogeneity of regression slopes. Where significant interactions were identified, these were retained in the model and probed using estimated marginal means to examine simple effects (i.e., group differences at each level of the covariate). Effect sizes are reported as partial eta squared (ηp^2^). Exploratory analyses were also performed to examine group differences in impairment rates for each binary sleep health dimension. Firth penalized logistic regression was performed with each sleep health dimension as the dependent variable and group, age, sex, and SES entered. Odds ratios and 95% confidence intervals are reported. Group mean differences in sleep behavior subdomains (Children’s Sleep Hygiene Scale) [33] were also examined with separate ANCOVAs.

For aim 2, a multiple linear regression analysis was conducted to examine the association between biopsychosocial predictor variables (neurodevelopmental traits composite, mental health composite, FSIQ, and PedsQL-NF1 pain) and the sleep health composite. Predictors were selected a priori based on theoretical relevance within a biopsychosocial framework [17]. Categorical variables (e.g., sex) were entered using dummy coding. All other variables were continuous. Variables were reversed (where appropriate) and standardized for regression. Spearman rho correlations were also performed to examine relationships between sleep health dimensions and biopsychosocial predictors. Spearman correlations were exploratory and presented using unadjusted *p*-values. For aim 3, a multiple linear regression was conducted to examine predictors (e.g., mental health composite, neurodevelopmental traits composite, sleep quality, sleep duration, and pain) of PedsQL Generic Total Score in the NF1 group. Prior to interpretation of the results, all assumptions of multiple linear regression were evaluated. Assumptions of logistic regression were evaluated using the Box–Tidwell procedure where possible. Visual inspection of scatterplots indicated that the assumptions of linearity and homoscedasticity were met. Examination of histograms and normal Q–Q plots of standardized residuals suggested that the residuals were approximately normally distributed. Independence of errors was supported by a Durbin–Watson statistic within acceptable limits. Multicollinearity was not observed, with variance inflation factors within acceptable ranges. Standardized residuals did not exceed ±3, indicating no evidence of problematic outliers. Standardized beta coefficients (β), unstandardized coefficients (B), confidence intervals, and R^2^ values are reported to describe effect sizes and model fit.

## 3. Results

### 3.1. Sample Characteristics

**Figure 2:**
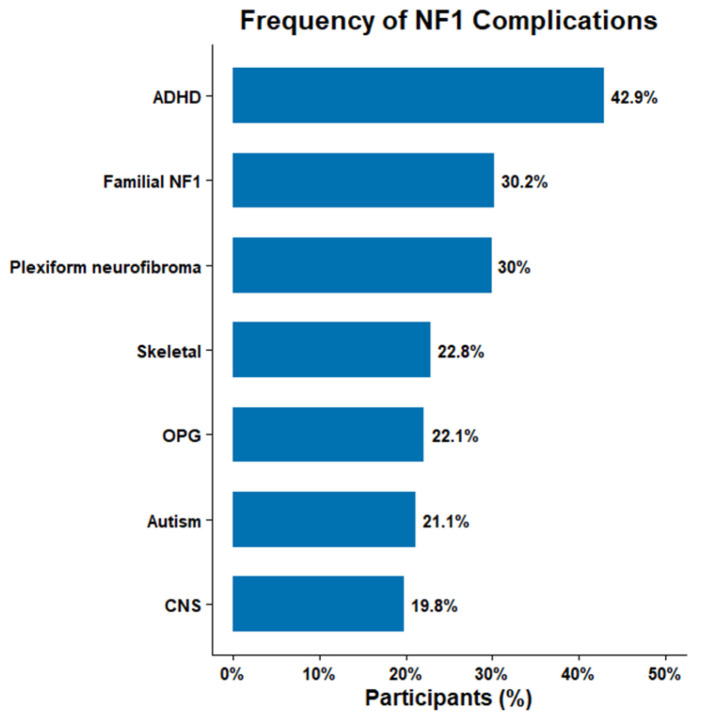
Frequency of NF1 complications within the NF1 cohort. ADHD = Attention deficit hyperactivity disorder. NF1 = Neurofibromatosis type 1. OPG = optic pathway glioma. CNS = Central nervous system abnormality, as outlined in methods.

Table 2 and Figure 2 show descriptive statistics for the groups, including percentages for dichotomous variables, and means and standard deviations for continuous variables. Corresponding descriptive statistics for participants with available sleep health data (n = 168) are provided in the Supplementary Materials (Table S2).

### 3.2. Group Differences in Sleep Health

**Figure 3:**
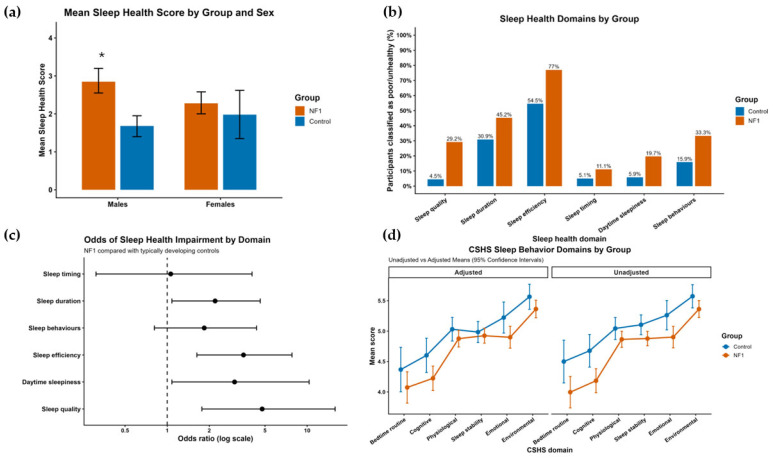
Group differences in Sleep Health and Sleep Health Dimensions: (**a**) Adjusted mean sleep health score by group and sex. Higher sleep health scores indicate poorer sleep health. Error bars represent 95% confidence intervals. * = *p* < 0.001. (**b**) Percentage of group classified as impaired (score of 1 rather than 0) on each sleep health dimension (unadjusted). (**c**) Forest plot of odds of sleep health impairment by dimension in children with NF1 compared with typically developing controls adjusted for age, sex and socioeconomic status. Values > 1 indicate higher odds of impairment in the NF1 group. Error bars represent 95% confidence intervals; crossing the dashed line indicates no significant difference (OR = 1). (**d**) Group mean scores and 95% confidence intervals for Children’s Sleep Hygiene Scale (Sleep Behavior) subdomains unadjusted and adjusted for sex, age, and socioeconomic status. Higher scores indicate better sleep behaviors.

An ANCOVA examining group differences in sleep health revealed a significant main effect of group (F(1,163) = 12.68, *p* < 0.001, ηp^2^ = 0.07), and a significant main effect of age (F(1,163) = 12.13, *p* = 0.001, ηp^2^ = 0.07). SES was not significant (*p* = 0.396). The interaction between group and sex was significant (F(1,163) = 5.26, *p* = 0.023, ηp^2^ = 0.03), indicating that group differences in sleep health were dependent on sex. Follow-up analyses probing interactions indicated that within males, participants in the NF1 group had significantly poorer sleep health scores compared with controls (mean difference = −1.18, CI [−1.68, −0.69], *p* < 0.001, ηp^2^ = 0.20). In contrast, there was no significant group difference in females (mean difference = −0.11, CI [−0.74, 0.51], *p* = 0.713, ηp^2^ = 0.002). These results suggest that sex moderated the relationship between group and sleep health, such that group differences are most pronounced in males but attenuated in females (Figure 3a).

### 3.3. Group Differences in Sleep Health Dimensions

Compared with their peers, children with NF1 showed a higher frequency of impairment across all sleep health dimensions, including sleep quality, sleep duration, sleep efficiency, sleep timing, daytime sleepiness, and sleep behaviors (Figure 3b). Following adjustment for age, sex, and SES, children with NF1 had greater odds of impairment in sleep quality, daytime sleepiness, sleep efficiency, and sleep duration compared with unaffected peers (Figure 3c).

Given that the sleep behavior dimension has multiple subdomains, we examined group differences for each subdomain (Figure 3d). Children with NF1 demonstrated poorer cognitive behaviors (e.g., cognitive hyperarousal before bed; *p* = 0.002), emotional behaviors (e.g., emotional state at bedtime such as worrying in bed (*p* = 0.011), and difficulties with bedtime routine (*p* = 0.020) when compared with unaffected children (Figure 3c).

### 3.4. Biopsychosocial Correlates of Sleep Health in NF1

The multiple linear regression model predicting sleep health in children and adolescents with NF1 was statistically significant (F(7,95) = 8.53, *p* < 0.001), explaining 38.60% of the variance in sleep health (R^2^ = 0.39), representing a large effect size (Table 3). Examination of individual predictors indicated that male sex was associated with poorer sleep health. Neurodevelopmental traits and pain were also significant predictors of poor sleep health in NF1. Age, mental health, SES, and FSIQ were not significantly associated with sleep health in children with NF1 after adjusting for other variables in the model.

### 3.5. How Do NF1 Complications Influence Sleep Health in NF1?

Given the relationship between poorer sleep health and elevated pain and neurodevelopment traits in NF1, further exploratory analyses were performed to examine whether sleep health differed between those with and without various NF1 physical complications. Children with familial (inherited) NF1 had poorer sleep health (*t* = −2.61, *p* = 0.01) compared with those with sporadic NF1 (de_novo *NF1* variation). There was no difference in sleep health between those with and without a skeletal complication, a plexiform neurofibroma, a CNS abnormality, or an OPG (all *p* > 0.05). In those with an ADHD diagnosis, sleep health was similar across those who took ADHD medication (*n* = 26) and those with ADHD who did not (*n* = 24, *p* > 0.05). In contrast, children who were taking other CNS medications (*n* = 22) had poorer sleep health (M = 3.01, SD = 1.28) compared with those who did not take CNS medications (*n* = 89, mean = 2.44 SD = 1.14, F = 4.31, *p* = 0.04).

### 3.6. Biopsychosocial Correlates of Individual Sleep Health Dimensions in NF1

**Figure 4:**
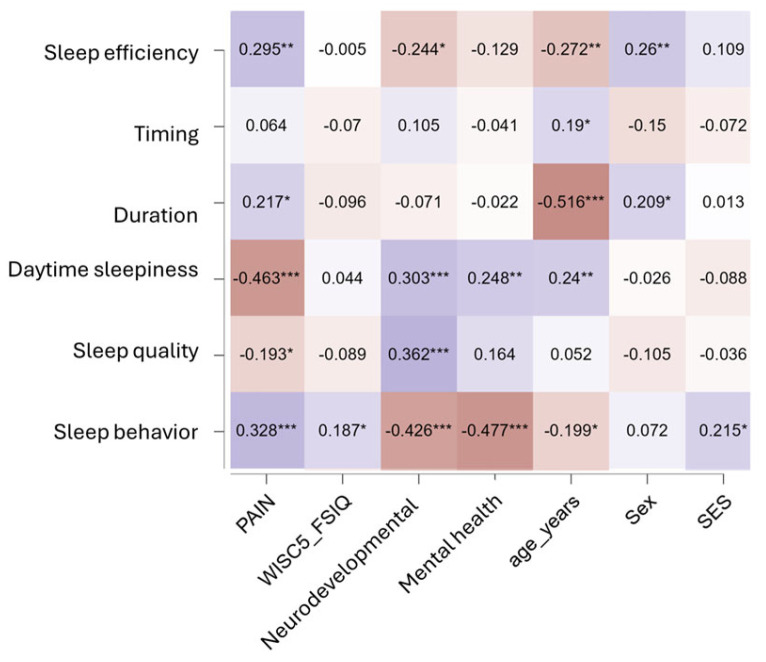
Heatmap showing Spearman rho correlations between biopsychosocial factors and individual sleep health dimensions in children with NF1. Colors indicate the strength and direction of correlation coefficients. Dark red represents strong negative correlations, dark purple represents strong positive correlations, and white indicates correlations close to zero. The color intensity is proportional to the magnitude of the correlation coefficient. * *p* < 0.05. ** *p* < 0.01 *** *p* < 0.001.

We next examined the relationship between biopsychosocial factors and individual sleep health dimensions in children with NF1 (Figure 4). Children with elevated pain had lower sleep efficiency (*p* = 0.002), greater daytime sleepiness (*p* < 0.001), and worse sleep behaviors (*p* < 0.001). Children with elevated neurodevelopmental traits had greater daytime sleepiness, poorer sleep quality, and poorer sleep behaviors (all *p* < 0.001). Elevated mental health was also associated with greater daytime sleepiness (*p* < 0.005) and worse sleep behaviors (*p* < 0.005). A higher FSIQ was associated with better sleep behaviors (*p* = 0.040). Male sex was associated with lower sleep efficiency (*p* = 0.006) and shorter sleep duration (*p* = 0.027).

### 3.7. Health-Related Quality of Life and Sleep in NF1

Children with NF1 had substantially lower parent-reported health-related quality of life (HR-QoL) compared with TD controls, even after adjusting for age, sex, and SES (adjusted mean 65.7 vs. 87.9; *p* < 0.001), representing a large effect (ηp^2^ = 0.25). A linear regression model was conducted to examine predictors of HR-QoL in children with NF1 (Table 4). The overall regression model was statistically significant, explaining 69.7% of the variance in HR-QoL, representing a large effect size (Table 4). Findings demonstrate that poorer mental health, elevated neurodevelopmental traits, greater pain, shorter sleep duration, and male sex predict poorer HR-QoL in children with NF1. Although univariate regression showed that both sleep quality and duration were strongly associated with HR-QoL, only sleep duration remained significant after adjustment for other predictors, indicating a unique contribution of sleep duration to HR-QoL in NF1.

## 4. Discussion

Using Buysse’s (2014) sleep health framework, which emphasizes the importance of multiple sleep characteristics for overall health and functioning, this study examined whether sleep health—including sleep timing, sleep quality, daytime sleepiness, sleep efficiency, sleep duration, and sleep-related behavior—is affected in children and adolescents with NF1 [15]. Compared with their typically developing peers, children with NF1 demonstrated poorer sleep health overall. While higher rates of impairment were evident across the six sleep health dimensions, impairments in sleep quality, daytime sleepiness, sleep efficiency, and sleep duration, derived from clinical cutoffs, emerged as the strongest contributors. Poor sleep quality was a predominant complaint occurring in approximately 30% of children with NF1, a rate five times higher than in unaffected children. Almost 78% of children with NF1 were found to have impaired sleep efficiency, suggesting most children with NF1 experience difficulties initiating and maintaining sleep based on objective assessment. As a result, nearly half the cohort was not getting a sufficient amount of sleep at night based on guidelines from the National Sleep Foundation [28].

The detrimental effects of poor sleep health, particularly insufficient sleep quantity and quality, on children’s cognitive, emotional, and overall functioning are well established [45,46]. The current findings extend this evidence to NF1, demonstrating that both sleep quality and quantity contributed to health-related quality of life (HR-QoL) in this population. Collectively, this evidence reinforces the clinical imperative to routinely assess sleep in NF1 care and the importance of proactive sleep health education for families. All patients with NF1 would benefit from screening of their sleep at routine clinic visits. If screening is positive, patients may benefit from a more detailed assessment of their sleep. Unfortunately, evidence-based recommendations for the screening and management of sleep disorders in patients with NF1 are limited due to the paucity of studies evaluating sleep disorders and therapeutic interventions in this population.

**Figure 5:**
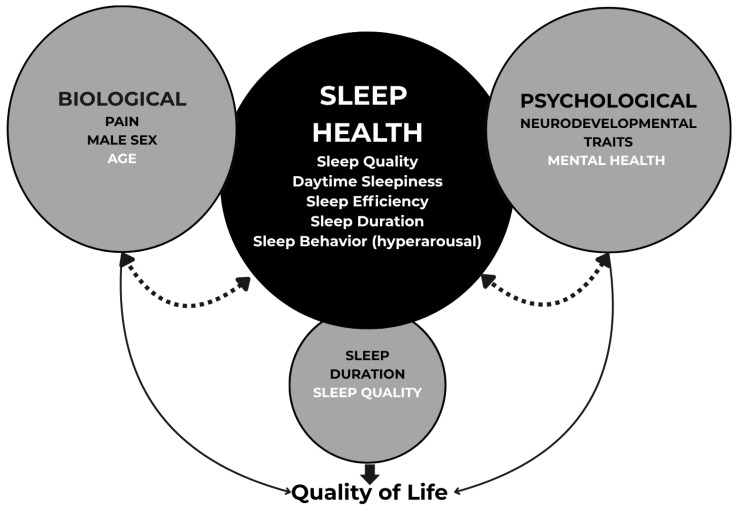
Conceptual biopsychosocial model of sleep health and health-related (QoL) in children and adolescents with neurofibromatosis type 1 (NF1). Poor sleep health in NF1 is conceptualized as a multidimensional construct encompassing sleep quality, sleepiness, sleep duration, sleep efficiency, and sleep behaviors (pre-sleep arousal). Associations between sleep health and biological and psychological factors are shown in black text (pain, sex, neurodevelopmental traits), with white text signifying a significant association found in univariate but not multivariate analyses (i.e., did not uniquely contribute within the full model). Dashed arrows are bidirectional, as the direction is not known from cross-sectional research. Sleep duration, mental health, neurodevelopmental traits, male sex, and pain independently contribute to QoL in NF1.

The current study identified a number of biopsychosocial correlates of sleep health in NF1 (Figure 5). In terms of biological factors, males experienced shorter sleep duration and lower sleep efficiency than females. Although sex differences in sleep are found in the general population [47], they are often linked to puberty [48], emerging in adolescence with mixed findings on which sex is impacted more. In this study, sex differences were independent of age, suggesting they manifest prior to puberty in NF1. Some have suggested that increased engagement with screens and gaming in males might contribute to sex differences in sleep in children in the general population [48]. We did not find a significant relationship between sex and sleep behaviors in NF1. Converging preclinical evidence from various NF1 animal models suggests a neurobiological basis for the sex-based findings of reduced sleep depth, lower arousal thresholds, and increased fragmentation in males. Male *Nf1*-null flies show shorter sleep duration compared with wild-type males, a difference not found in females [6]. Interestingly, sex differences in neuronal dopamine, a neurotransmitter that regulates the generation of sleep–wake states [49], have also been shown in studies involving *Nf1*-conditional knock out mice, with males exhibiting decreased hippocampal dopamine compared to females [50]. Despite this, sex is typically not found to be a significant determinant of the behavioral phenotype in NF1 [51]. Taken together, these findings suggest that boys with NF1 represent a group at particular risk for poor sleep health and highlight the need for future studies to consider sex-specific mechanisms that may modulate or influence sleep circuitry and arousal systems in NF1.

The strongest correlate of sleep health in NF1 was elevated neurodevelopmental traits, specifically ADHD and autism. More specifically, children with elevated neurodevelopmental traits experienced more difficulties with sleep behaviors, poorer sleep quality, and reduced sleep efficiency. This aligns with recent evidence that has shown an ADHD diagnosis to be associated with sleep disturbance and sleep impairment (using the PROMIS) in youth with NF1 [20]. It is well known that children and adolescents with ADHD (without NF1) share similarities in their sleep phenotype, including more frequent sleep disturbances, lower sleep efficiency, poorer sleep quality, and greater daytime sleepiness than neurotypical peers [52]. The established link between ADHD and specific sleep disorders, including restless legs syndrome and obstructive sleep apnoea [52], provides a framework to interpret the subjective reports and actigraphic abnormalities in ADHD, highlighting the need for further research investigating these sleep disorders in NF1 and whether they play a role in the sleep phenotype of NF1.

The biological mechanisms explaining the link between sleep and neurodevelopmental traits in NF1 are likely complex and are yet to be identified. NF1 is implicated in several Ras pathways, including RAF/MEK/ERK [1], Akt/mTOR, and cAMP [53], as well as GABAergic and dopaminergic signaling processes. These pathways have been implicated in an increased risk for neurodevelopmental outcomes in NF1 animal and human studies [53], as well as sleep regulation and circadian functioning in animal models of NF1 [6,9,10,54,55,56]. Emerging research, although still in its early stages, suggests that alterations in neuroplasticity and an imbalance in excitation–inhibition may be one mechanism contributing to the increased risk of sleep and circadian disruption in children with neurodevelopmental conditions [52]. Future research focusing on clarifying shared neurobiological pathways of sleep and neurodevelopment, including arousal–regulation pathways, is needed in humans with NF1.

Our study found that sleep health was similarly impaired across children with NF1, irrespective of the physical burden of the condition, including skeletal abnormalities, plexiform neurofibromas, or CNS abnormalities. This aligns with preclinical evidence showing that zebrafish with one wild-type *NF1* allele exhibit disrupted sleep at a developmental stage prior to the establishment of manifestations such as tumors [9] and may suggest that poor sleep health in NF1 is not secondary to NF1 physical complications.

Although overt physical NF1 complications were not associated with sleep health in this cohort, pain remained an important correlate of sleep difficulties. One possible explanation for why sleep was associated with pain but not the physical manifestations of NF1 could be that pain reported in this study arises from mechanisms not captured by these broad complication groupings, such as headache pain, restless leg pain, or altered nociceptive processing. Whatever the cause of pain in these children, we found elevated subjective pain was associated with lower sleep efficiency, shorter sleep duration, greater daytime sleepiness, and poorer sleep behaviors in children with NF1. These associations may reflect a pathway whereby pain-related distress and pre-sleep cognitive/emotional arousal (e.g., worrying about sleep) contribute to difficulties initiating and/or maintaining sleep, resulting in shorter sleep and increased sleepiness the following day. Given the cross-sectional design, however, we cannot determine the directionality of these relationships. Indeed, a systematic review that included different pain conditions in paediatric populations supports a bidirectional relationship between sleep and pain [57]. Various mechanisms have been proposed to explain this bidirectional relationship, including disruption to endogenous pain modulation, where pain thresholds are lowered due to insufficient sleep [19]. For example, studies that have manipulated sleep in healthy individuals have shown that partial sleep deprivation can impair endogenous pain modulation. Restriction of sleep over a 3-week period led to increases in pain, decreased pain habituation, and increased temporal summation, suggesting exposure to chronic insufficient sleep may increase vulnerability to chronic pain by alterations in pain sensitization [58]. Interestingly, evidence from the minipig model of NF1 (*Nf1*+/^ex42del^) suggests that there may be biologically linked pathways to pain and sleep, with nociceptive signaling linked to pain behaviors and sleep quality [11]. Overall, our findings indicate that pain is an important correlate of poorer sleep health in NF1, highlighting the importance of integrated assessment and management of pain and sleep in NF1.

The finding of poorer sleep health among children with familial NF1 should be interpreted cautiously. Our data cannot determine the underlying reasons for this observation. It is possible that unmeasured factors, including parental mental health or stress, neurodevelopmental comorbidities, shared familial environmental influences, or parent medical challenges contribute to this finding. However, this observation requires confirmation in other cohorts.

Our study has several clinical implications and raises some important avenues for future research. Targeting pre-sleep arousal, particularly in those with NF1 + ADHD/autism, may provide a potential treatment route for poor sleep efficiency (especially sleep onset issues). Cognitive behavioral therapy for insomnia (CBT-I) is a structured evidence-based approach that has been shown to reduce pre-sleep arousal in adults with insomnia [59] and has been shown to improve sleep quality and onset in adolescents, with emerging evidence in school-aged children [60]. Behavioral sleep interventions that focus on sleep hygiene, progressive muscle relaxation, and stimulus control are also a promising avenue for future research and may assist those with elevated pain or difficulties with sleep behaviors. Indeed, a number of systematic reviews support the use of behavioral sleep interventions in children [61] as well as in those affected by ADHD, with moderate effect sizes reported [62]. The absence of any published trials targeting sleep disruption in NF1 highlight an urgent and unmet clinical need, particularly given the functional consequences for HR-QoL identified in the current study.

An important limitation of the current study is that the threshold used to define impaired sleep timing irregularity (i.e., > 1 SD in midpoint sleep variability) and daytime sleepiness was empirically derived rather than based on an established paediatric clinical cutoff. Although this approach is consistent with efforts to operationalize multidimensional sleep health [30], it may have reduced sensitivity to more subtle between-group differences in sleep timing irregularity, which have been shown previously [14], and thus should be interpreted as indicating relative rather than clinically validated irregularity.

## 5. Conclusions

In conclusion, sleep disruption is markedly more common in children and adolescents with NF1 than their neurotypical peers, supporting the value of a multidimensional sleep health framework in this population. Sleep difficulties in NF1 were not limited to a single domain but were evident across domains, including sleep quality, sleep efficiency, sleep duration, and sleep-related behaviors, highlighting the broad and clinically meaningful nature of sleep disruption. The findings also suggest that poorer sleep health in NF1 is associated with multiple interacting biopsychosocial factors, with male sex, elevated neurodevelopmental traits, and pain emerging as the strongest correlates.

Sleep duration uniquely contributed to health-related quality of life beyond established predictors, indicating that sleep may represent a modifiable and under-recognized treatment target in NF1. Together, these findings support the routine assessment of sleep as part of comprehensive clinical care for children and adolescents with NF1 and underscore the need for future longitudinal and intervention studies to clarify mechanisms, identify children at greatest risk, and determine whether improving sleep health can enhance broader developmental and functional outcomes.

## Figures and Tables

**Table 1:** Dimensions used to construct multidimensional sleep health composite.

Dimension	Measure	Cut Points
Efficiency	Actigraphy sleep efficiency	0 ≥ 85%: good efficiency; 1 < 85%: poor efficiency [29]
Duration	Actigraphy average total sleep time (TST) hours	0 TST ≥ 9 and child 6–14 years or TST ≥ 8 and 14–17 years: sufficient sleep [28] 1 TST outside this range: insufficient sleep
Sleepiness	Sleep Disturbance Scale for Children [31] Disorders of Excessive Somnolence	0 ≤ 10: no/low daytime sleepiness 1 > 10: elevated daytime sleepiness
Quality	Pittsburgh Sleep Quality Index (PSQI) [32] Sleep Quality Component Score	0 = responded good or very good 1 = responded bad or very bad
Timing	Actigraphy-derived average standard deviation (SD) of sleep midpoint	0 ≤ 1 SD (1 h): regular timing 1 > 1 SD (1 h): irregular timing
Behavior	Children’s Sleep Hygiene Scale Total [33]	0 ≥ 1 SD: good behavior; 1 < 1 SD: poor behavior

**Table 2:** Demographic and descriptive variables.

Variable	Controls	NF1	Effect Size	95% CI for Effect Size
Sex (males %)	63.8	50.4	ns	
Age, y (mean, SD)	10.0 ± 2.81	10.67 ± 2.71	0.22	−0.17, 0.52
Body mass index (mean, SD)	17.86 ± 4.40	17.66 ± 3.78	0.04	−0.02, 0.33
SES (median, IQR)	87.00 ± 22.00	56.00 ± 51.00	−0.44 ***	−0.56, −0.29
FSIQ (mean, SD)	109.20 ± 13.05	86.32 ± 14.01	−1.67 ***	−2.00, −1.33
Neurodevelopmental traits (mean, SD)	48.86 ± 8.31	63.39 ± 13.20	1.23 ***	0.91, 1.55
Mental health (mean, SD)	47.83 ± 6.42	55.27 ± 11.02	0.76 ***	0.46, 1.07
PedsQL Generic total (mean, SD)	87.13 ± 10.13	65.17 ± 19.85	−1.32 ***	−1.65, −0.99
PedsQLNF1 Pain (mean, SD)	-	78.57 ± 20.76	-	-
SDSC DOES raw score (median, IQR)	6.00 ± 3.00	7.00 ± 3.00	0.21 ^a^ *	0.05, 0.37
CSHS total score (mean, SD)	5.00 ± 0.51	4.69 ± 0.59	−0.55 ***	−0.86, −0.24
Sleep health composite (mean, SD)	1.79 ± 1.09	2.57 ± 1.19	0.67 ***	0.34, 1.00

PedsQL = The Paediatric Quality of Life Inventory. SDSC DOES = Sleep Disturbance Scale for Children, Disorders of Excessive Somnolence Score. CSHS = Children’s Sleep Hygiene Scale. Effect sizes are Cohen’s d unless non-parametric (^a^) and then rank-biserial is reported * *p* < 0.05. *** *p* < 0.001.

**Table 3:** Univariate and multivariate linear regression analysis predicting sleep health in NF1.

	Univariate			Multivariate		
Predictor	β	95% CI	*p*	β	95% CI	*p*
Age	−0.27	−0.46, −0.09	0.004	−0.17	−0.35, 0.00	0.054
Sex	−0.25	−0.43, −0.07	0.008	−0.25	−0.41, −0.08	0.003 **
SES	−0.12	−0.31, 0.07	0.201	−0.08	−0.24, 0.08	0.317
FSIQ	−0.10	−0.29, 0.09	0.301	0.00	−0.16, 0.17	0.988
Mental health	−0.30	−0.47, −0.12	0.001	−0.04	−0.23, 0.16	0.692
Neurodevelopmental traits	−0.46	−0.61, −0.28	<0.001	−0.35	−0.51, −0.15	<0.001 ***
Pain	−0.38	−0.57, −0.20	<0.001	−0.21	−0.41, −0.02	0.028 *

Model statistics: N = 103, R^2^ = 0.39, adjusted R^2^ = 0.34, F(7,95) = 8.53, * *p* < 0.05 ** *p* < 0.01 *** *p* < 0.001.

**Table 4:** Univariate and multivariate linear regression analysis predicting HR-QoL in NF1.

		Univariate			Multivariate		
Predictor	N	β	95% CI	*p*	β	95% CI	*p*
Age	119	0.19	0.00, 0.37	0.036 *	−0.01	−0.15, 0.13	0.885
Sex	119	0.16	−0.02, 0.34	0.088	0.13	0.00, 0.26	0.045 *
SES	119	0.05	−0.13, 0.23	0.619	−0.09	−0.21, 0.03	0.147
Mental health	119	0.61	0.47, 0.78	<0.001 *	0.32	0.18, 0.48	<0.001 ***
Neurodevelopmental traits	118	0.63	0.49, 0.78	<0.001 *	0.37	0.21, 0.51	<0.001 ***
Sleep duration	103	0.32	0.13, 0.51	0.001 *	0.17	0.23, 0.31	0.024 *
Sleep quality	117	0.34	0.17, 0.52	<0.001 *	−0.02	−0.16, 0.13	0.810
Pain	115	0.60	0.44, 0.74	<0.001 *	0.32	0.17, 0.46	<0.001 ***

Model statistics: N = 98, R^2^ = 0.69, adjusted R^2^ = 0.66, F(8,82) = 23.57, * *p* < 0.05 *** *p* < 0.001.

## Data Availability

The dataset used in this study can be accessed upon request. Following an embargo period, the data will be available (on approximately 1 January 2029) at the Sage NF Portal [https://nf.synapse.org/].

## Supplementary Materials

The following supporting information can be downloaded at: https://www.mdpi.com/article/10.3390/cancers18142366/s1, Table S1: Sleep health dimension measures and coding details, Table S2: Demographic and descriptive variables of participants with available sleep health data.

## Abbreviations

The following abbreviations are used in this manuscript:

NF1	Neurofibromatosis type 1
QoL	Quality of life
TD	Typically developing
HR	Health-related
ADHD	Attention deficit hyperactivity disorder
GAP	GTPase-activating protein
CNS	Central nervous system
SES	Socioeconomic status
PedsQL	Pediatric Quality of Life
BASC	Behavior Assessment System for Children
CONSORT	Consolidated standards of reporting trials
TST	Total sleep time
SD	Standard deviation
PRS	Parent rating scale
WISC	Wechsler Intelligence Scale for Children
FSIQ	Full-Scale Intelligence Quotient
SRS-2	Social Responsiveness Scale Version Two
OPG	Optic pathway glioma
CSHS	Children’s Sleep Hygiene Scale
SDSC	Sleep Disturbance Scale for Children
DOES	Disorders of excessive sleepiness
CI	Confidence interval
RAF	RAF kinase
MEK	Mitogen-activated protein kinase
ERK	Extracellular signal regulated kinase
mTOR	Mechanistic target of rapamycin
AC	Adenylyl cyclase
cAMP	Cyclic adenosine monophosphate
GABA	Gamma-aminobutyric acid
CBT-I	Cognitive behavioral therapy for insomnia
