# Supplementary material for: Sleep Health and Quality of Life in Children and Adolescents with NF1: A Biopsychosocial Perspective
Source: Cancers (Basel). 2026 Jul 22;18(14):2366. doi: 10.3390/cancers18142366 (PMC13406831; doi:10.3390/cancers18142366)
Supplement: Supplementary file 1 [file cancers-18-02366-s001.zip › cancers-4375658-supplementary.pdf]

Table S1. Sleep Health Dimension Measures and Coding Details

Sleep Health Dimension	Coding Details
Sleep duration	Average total of hours of sleep based on actigraphy over 1 week. The average sleep duration was then classified as adequate sleep and short sleep based on the National Sleep Foundation recommendations. According to these recommendations children aged between 6-13 years require at least 9 hours of sleep a night and adolescents aged 14–17 years require at least 8 hours. Participants were coded 0 if they met the requirement and 1 if they fell short.
Sleep efficiency	This was derived from actigraphy and was calculated as the ratio of time spent in asleep versus time in bed, multiplied by 100 (expressed as a percentage). The average sleep efficiency score across the seven nights was dichotomized into low sleep efficiency (<85 percent; coded as 1) and normal (≥ 85%; coded as 0) based on clinical recommendations.
Sleep quality	Pittsburgh Sleep Quality Index (PSQI) amended for paediatric populations was completed by the caregiver. The PSQI assesses seven domains, some of which overlap with other sleep health dimensions. As such, we used the first component of the PSQI which assesses sleep quality. We classified sleep quality using the following criteria: fairly bad and very bad were coded as poor sleep quality (coded as 1) and good or very good was classified as good sleep quality (coded as 0).
Sleep timing	Sleep Timing was assessed by actigraphy derived standard deviation (SD) of sleep midpoint across seven days. The average SD was classified as irregular (>1SD i.e. > 1 hour, coded as 1) or regular (≤ 1 SD, coded as 0).
Daytime sleepiness	Sleepiness was assessed using the well validated carer questionnaire the Sleep Disturbance Scale for Children. The disorders of excessive somnolence (DOES) scale was used to assess sleepiness. This subscale provides a raw score. Participants were dichotomized according to the measure's cutoff as elevated sleepiness (raw score > 10, coded as 1) and no/low sleepiness (raw score ≤ 10; coded 0).
Sleep Behavior	The Children's Sleep Hygiene Scale (CSHS) divides sleep-related behaviors into six subscales as well as a total score. The subscales include Physiological (behaviors that affect body's physical readiness for sleep such as caffeine intake, hunger/thirst), Cognitive (hyperarousal), Emotional (emotional state at bedtime), Sleep Environment (quality of child's sleep environment), and Sleep Stability (consistent sleep schedule). Higher scores indicate better sleep behaviors. As there is no normative data for this measure we used the control group mean as a cutoff and classified all participants as either having poor total sleep behaviors (≤ 1SD below the control mean; code 1) or good total sleep behaviors (all other scores, code 0).

Table S2. Demographic and Descriptive Variables of participants with available sleep health data

Variable	Controls (n=56)<br>Mean (SD)	NF1 (n=112)<br>Mean (SD)
Sex (males %)	62.5	49.1
Age, y	10.21 ± 2.78	10.55 ± 2.67
Body mass index	17.80 ± 4.15	17.74 ± 4.00
SES	78.71 ± 20.49	56.19 ± 29.65***
FSIQ	109.12 ± 12.71	86.21 ± 13.91***
Neurodevelopmental traits	48.93 ± 8.59	63.81 ± 13.61***
Mental health	47.97 ± 6.88	54.32 ± 11.02***
PedsQL Total	87.53 ± 9.49	65.34 ± 19.62***
PedsQLNF1 Pain	-	78.66 ± 20.46

PedsQL = The Paediatric Quality of Life Inventory *** $p < .001$
